# Defining the S-Glutathionylation Proteome by Biochemical and Mass Spectrometric Approaches

**DOI:** 10.3390/antiox11112272

**Published:** 2022-11-17

**Authors:** Xiaolu Li, Tong Zhang, Nicholas J. Day, Song Feng, Matthew J. Gaffrey, Wei-Jun Qian

**Affiliations:** Biological Sciences Division, Pacific Northwest National Laboratory, Richland, WA 99354, USA

**Keywords:** redox, redox proteomics, post-translational modification, S-glutathionylation, mass spectrometry

## Abstract

Protein S-glutathionylation (SSG) is a reversible post-translational modification (PTM) featuring the conjugation of glutathione to a protein cysteine thiol. SSG can alter protein structure, activity, subcellular localization, and interaction with small molecules and other proteins. Thus, it plays a critical role in redox signaling and regulation in various physiological activities and pathological events. In this review, we summarize current biochemical and analytical approaches for characterizing SSG at both the proteome level and at individual protein levels. To illustrate the mechanism underlying SSG-mediated redox regulation, we highlight recent examples of functional and structural consequences of SSG modifications. Finally, we discuss the analytical challenges in characterizing SSG and the thiol PTM landscape, future directions for understanding of the role of SSG in redox signaling and regulation and its interplay with other PTMs, and the potential role of computational approaches to accelerate functional discovery.

## 1. Introduction

Cellular redox homeostasis is a dynamic and controlled process that continuously balances the generation and removal of electrophiles (e.g., reactive oxygen species, or ROS) and nucleophiles (antioxidant defense systems) under the physiological steady state [1]. At the homeostatic level, ROS such as superoxide anion radical (O_2_^•−^), hydrogen peroxide (H_2_O_2_), or hydroxyl radical (HO^•^) are produced endogenously during normal aerobic metabolism and play an essential role in cell signaling and regulation [2,3]. A marked increase in ROS production under various stimuli can lead to the development of pathological conditions [4]. To maintain redox homeostasis, multiple types of antioxidants act to scavenge ROS, including antioxidation enzymes (e.g., superoxide dismutase, catalase, glutaredoxin, and thioredoxin), low-molecular weight antioxidants (e.g., glutathione and coenzyme Q), and dietary antioxidants (e.g., vitamin C) [5,6]. Among them, the highly abundant (~1–10 mM in cells) tripeptide glutathione (L-γ-glutamyl-L-cysteinylglycine, GSH) is a key component of the intracellular redox buffering system [7]. Two free GSH molecules can be reversibly oxidized to GSSG by forming a disulfide bond. The overall GSH/GSSG ratio in cells is typically greater than 30:1 or even ≥100:1 to provide a strong reducing cellular environment for normal physiological activities and tightly regulated to maintain a proper cellular redox state. A significant decrease in the GSH/GSSG ratio has been reported during oxidative stress with either over-production of ROS and/or deficient capacity of the antioxidant systems [8,9].

**Figure 1:**
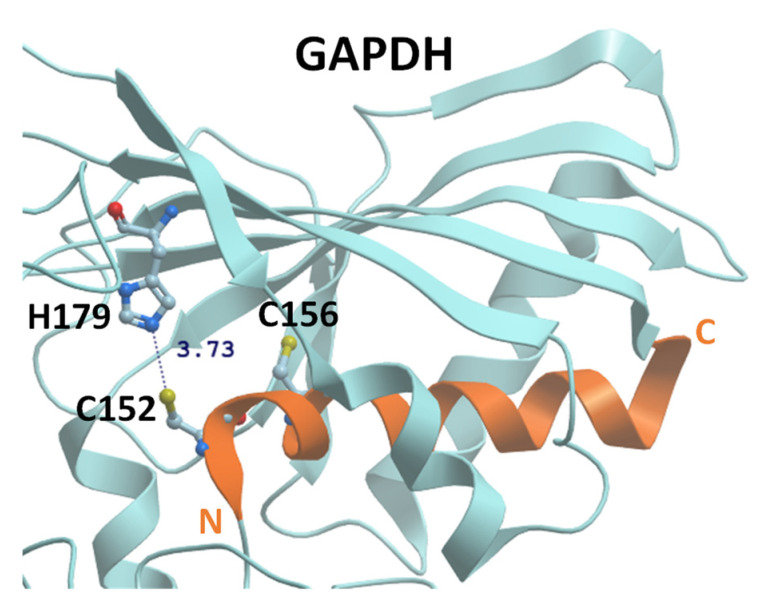
Electrostatic microenvironment lowers cysteine thiol pK_a_ and stabilizes thiolate anion in human GAPDH (PDB: 6yne).

Protein cysteine (Cys) thiols have been increasingly recognized for their critical role in redox-dependent signaling and regulation through a diverse set of post-translational modifications (PTMs), including protein S-glutathionylation (SSG). The reactivity of protein Cys thiols is largely dependent on the pK_a_ of their thiol group, as well as the protein microenvironment where the thiol group resides. While many protein Cys thiols retain pK_a_ > 8, and thus, are almost completely protonated (SH) and stable under physiological conditions [10,11], reactive cysteines exhibit relatively low pK_a_ and can easily form highly reactive thiolate anions (S^−^) under neutral pH. The low thiol pK_a_ can be attributed to neighboring amino acids with positive charges (e.g., arginine, lysine, histidine) or those that promote hydrogen bonds (e.g., serine, threonine, tyrosine) in protein three-dimensional structures [12,13]. It has also been reported that in the dipoles of α-helices, N-termini facing towards Cys residues lowers the thiol pK_a_ and stabilizes thiolate anions [14]. For instance, the well-known redox-sensitive Cys site (Cys-152) in human glyceraldehyde-3-phosphate dehydrogenase (GAPDH) is vicinal to His-179 and has an estimated pK_a_ of 6.03 [15]. An X-ray crystal structure (PDB: 6yne) shows that C152 is located at the N-terminus of an α-helix that can stabilize the thiolate anion (Figure 1). SSG modification of this highly conserved catalytic Cys site is reported in an array of organisms and reversibly inhibits enzymatic activity [16,17,18,19]. The accessibility of Cys thiols to various molecules or solvents (i.e., whether the Cys thiol is buried in protein folds or exposed to the surfaces) is another factor determining Cys reactivity. For instance, the surface-located active site Cys-72 of recombinant human thioredoxin has greater susceptibility to form a SSG modification and was found to be the only site alkylated by iodoacetamide under non-denaturing conditions [20].

Reactive Cys thiols can be reversibly oxidized to several forms of PTMs, including S-sulfenylation (SOH), S-nitrosylation (SNO), SSG, S-polysulfidation (SS_n_H, *n* ≥ 1), and disulfides (S-S). These PTMs can lead to changes in protein function and conformation, subcellular location, and protein interaction with proteins or small molecules. Among the PTMs, SSG is recognized as one of the most prevalent forms with significant biological relevance [21,22]. Initially, SSG was considered to serve a protective role to avoid over-oxidation of thiols to irreversible forms, such as S-sulfinylation (SO_2_H) and S-sulfonylation (SO_3_H). However, the regulatory and signaling roles of SSG have been increasingly established in recent years. With an expanding list of protein targets being discovered, SSG has been observed as being broadly involved in the regulation of gene expression, signaling, metabolism, cell death and survival, as well as protein folding and degradation [21,23]. The significance of SSG in the physiology and pathology of various human diseases, such as neurodegenerative, cancer, lung, cardiovascular, and other inflammatory diseases, have also been extensively reported and reviewed [9,22,24,25,26].

**Figure 2:**
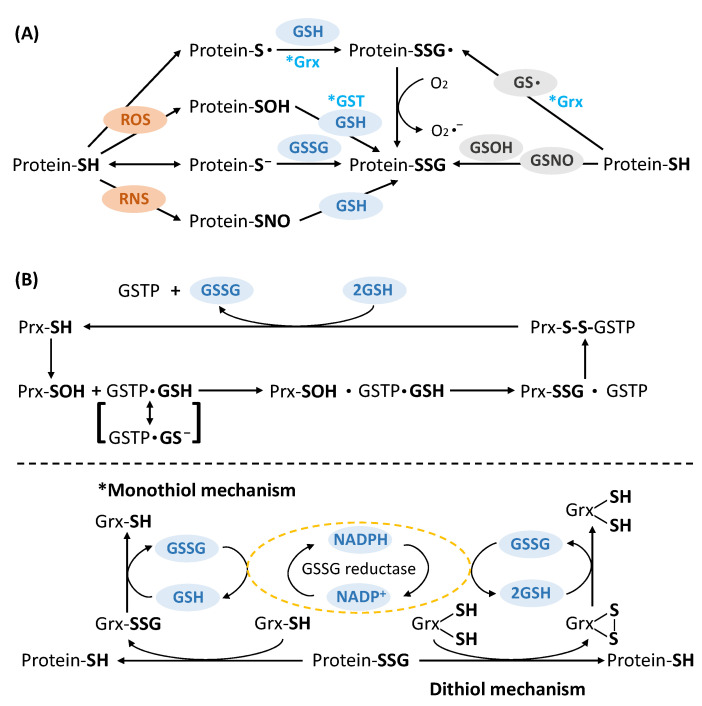
(**A**) Potential molecular mechanisms of protein glutathionylation. (**B**) Protein glutathionylation (GSTP, adapted from [41]) and de-glutathionylation by enzymatic reaction (Grx).

Given the abundant cellular availability of GSH and GSSG, reactive cysteines can form SSG via a diverse set of non-enzymatic or enzymatic mechanisms. Briefly, SSG can be formed via several ROS-mediated pathways (Figure 2A), including: (1) thiol-disulfide exchange between protein thiols and GSSG; (2) GSH directly reacting with unstable oxidized sulfhydryl intermediates (protein SOH and thiyl radical S^•^) induced by ROS; (3) protein thiols reacting with GSOH or GS^•^ [27,28,29,30,31,32]. Nitric oxide (NO)-induced SSG is another proposed mechanism via secondary generation of reactive nitrogen species (RNS). Nitrosoglutathione (GSNO) was reported to induce both SNO and SSG in many in vitro studies on papain, GAPDH, creatine kinase, actin, c-Jun, etc. [33,34]. Furthermore, SSG formation can be facilitated by enzymatic processes. Glutathione S-transferase (GST, primarily GST-pi or GSTP) has been the most studied enzyme promoting SSG formation on a wide array of proteins [35,36,37,38,39]. GSTP is proposed to bind GSH and lower its thiol pK_a_, thus forming a GS^−^ anion. GS^−^ can, then, be transferred to receptive cysteines in protein targets [40]. Other studies have demonstrated that oxidized 1-cys peroxiredoxin (Prx-SOH) can be reactivated by GSTP, which transfers GSH to facilitate Prx-SSG via formation of a GSTP(GSH)-Prx-SOH complex, followed by inter-disulfide formation and subsequent reduction (activation) of 1-CysPrx [41,42] (Figure 2B).

While the formation of SSG can occur via non-enzymatic or enzymatic mechanisms, de-glutathionylation is usually catalyzed by enzymes to precisely control the reversal process. Current known enzymes include glutaredoxin (Grx), sulfiredoxin (Srx), GST-omega (GSTO), and thioredoxin (Trx) [43,44,45,46]. Grx is the major player in reducing SSG to free thiols in mammalian cells by attacking the glutathionyl sulfur of SSG and forming SSG on its active-site cysteine of the CXXC motif. Grx-SSG can then be reduced by free GSH via thiol-disulfide exchange (because of the low pK_a_ of the Grx active-site cysteine), while the product GSSG is then reduced by GSSG reductase and NADPH (Figure 2B, monothiol mechanism) [47]. On the other hand, it was also proposed that both cysteines in the active site of Grx can form a disulfide to reduce SSG on protein targets (dithiol mechanism). The monothiol mechanism is recognized as the prevalent mechanism [48]. GSTO shares similar functional structure to Grx, which performs deglutathionylation through a similar mechanism using GSH as co-substrate. By contrast, Srx and Trx were initially characterized to reduce protein-SO_2_H and disulfides (and other redox PTMs), respectively, but show broader substrate specificity [49]. The role and mechanism of Srx- and Trx-catalyzed deglutathionylation is still not fully understood.

Our knowledge of SSG modifications is largely dependent on analytical approaches for the characterization of this modification. Significant advances in biomedical and mass spectrometry (MS)-based proteomics approaches have transformed our understanding of the SSG proteome. While biochemical methods such as immunoblotting with anti-GSH antibody are generally easy to implement, MS-based proteomics has become an indispensable tool for enabling broad site-specific quantification of SSG modifications and for greatly expanding our knowledge of the SSG proteome. Herein, we present an overview on both biochemical methods and MS-based quantitative proteomics approaches for characterizing SSG with a focus on recent advances in SSG proteome characterization. We also showcase novel discoveries on the SSG-dependent regulation of protein functions. Lastly, limitations of current approaches and future perspectives on the SSG proteome characterization, including structural and functional aspects, are discussed.

## 2. General Strategies for the Detection of Protein S-Glutathionylation

**Figure 3:**
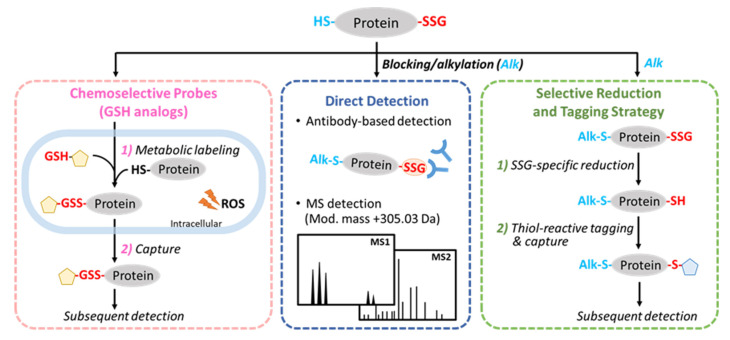
General strategies for detecting protein glutathionylation.

There are three general strategies for SSG detection (Figure 3). The first one is to directly detect the SSG moieties (through preserving endogenous SSG) by either affinity reagents, such as anti-GSH antibodies, or MS. While this strategy seems to be straightforward, it has been generally challenging for such direct detection due to the lack of specificity of affinity reagents and the labile nature of the modification for MS detection. The second strategy involves selective reduction of SSG back to a free thiol followed by thiol-reactive tagging to enable subsequent detection. In this case, the protein cysteine thiols (SH) need to be initially blocked by alkylation reagents such as *N*-ethylmaleimide (NEM). Then, SSG is selectively reduced back to a free thiol (e.g., by Grx), and the nascent SH is tagged by various chemical approaches or enriched by a thiol-reactive resin [50], to facilitate subsequent detection either using biochemical assays or MS. The third strategy utilizes a chemoselective GSH probe (GSH analog) to metabolically label reactive protein Cys thiols in situ and form protein-SSG with a tag, which can then be enriched and detected. One should note that the third strategy detects “artificially” formed SSG with the GSH analogs, but not endogenous SSG modifications.

Regardless of the strategies, critical to successful analysis of SSG is the preservation of the endogenous protein thiol redox state and prevention of potential oxidative artifacts during sample processing. Thiol-reactive alkylation reagents such as NEM and iodoacetamide (IAM) are commonly used to block free thiols to minimize potential oxidation, thiol-disulfide exchange (e.g., between reactive thiols and SSG), and disulfide scrambling during sample processing. In the case of SSG, transient thiol PTMs such as SOH and SNO, as well as disulfides, could contribute to a false positive identification [51,52,53]. Acidic pH is also useful to protonate reactive thiols and stabilize the redox proteome. For example, trichloroacetic acid (TCA) precipitation is often used during cell lysis and protein extraction, where a buffer pH around 6–7 can be used in certain processing steps [54,55,56]. After blocking, protein SSG can be subjected to various strategies to facilitate the detection. In the following sections, we will describe various detection methods in detail.

## 3. Methods for Characterizing Protein S-Glutathionylation

### 3.1. Direct Detection

Radiolabeled (^35^S-) GSH was one of the earliest tools used for direct detection of SSG [20,57,58]. To analyze in vivo SSG, protein synthesis is first blocked by preincubating cells with cycloheximide and followed by supplement of ^35^S-cysteine in cell culture to radioactively label the intracellular GSH pool. Induced by oxidative stress, the synthesized ^35^S-GSH is employed to form radiolabeled SSG, which can be extracted and visualized on a non-reducing SDS-PAGE gel using autoradiography and phosphor imaging technology (after drying the gel or transferring to nitrocellulose). Using this method, a number of proteins were identified as modified by GSH in vivo, such as GAPDH [59], actin [60], protein kinase C-α [61], and thioredoxin [20]. This method was further coupled with peptide mass fingerprinting to identify more proteins in mammalian cells, yeast, and algae [57,58,62]. However, this method has several limitations, including the disturbed cell physiology resulting from the need to block protein synthesis, potential non-specific signals (e.g., radiolabeled cysteinylated proteins), and the inability to distinguish individual SSG sites.

Another type of popular tool for direct detection of SSG is through Western immunoblotting of glutathionylated proteins on one-dimensional (1D) or two-dimensional (2D) non-reducing gels using anti-GSH antibodies [63,64]. Similar to radiolabeled GSH, the antibody method has limited specificity and sensitivity and is unable to distinguish individual SSG sites within a target protein, which may have different functional consequences. Nevertheless, this method proves to be a useful tool to evaluate global SSG level changes. In conjunction with protein-specific antibodies, it also allows for the quantification of SSG of targeted proteins in complex samples [65,66,67].

Besides the radiolabeled and antibody approaches, mass spectrometry can also directly detect the SSG moiety on proteins or peptides. For example, top-down proteomics has been applied to identify sarcomeric protein PTMs, including SSG [68,69]. By detecting the glutathione conjugation (+305.03 Da) on proteoforms, SSG on troponin I (TnI) was identified as a potential marker that increases with aging. Alternatively, we recently applied bottom-up MS-based global proteome profiling to directly detect thiol PTMs without enrichment [70]. In this approach, proteins were extracted by blocking free thiols and polysulfides (while other thiol PTMs were preserved) and then digested. The resulting peptides were then subjected to fractionation and proteome profiling. In the end, only 81 unique Cys sites with SSG were identified, illustrating the challenge of direct SSG detection by MS proteomics.

### 3.2. Selective Reduction and Tagging Approaches

Given the challenges in direct detection of SSG, alternative methods are necessary to enable effective characterization. The selective reduction and tagging strategy has become the most applied approach for enabling enrichment and SSG measurement. This strategy was largely built upon the concept of differential alkylation, which was first introduced in the biotin switch technique (BST) for protein SNO detection [71]. BST was later modified to profile other redox PTMs including SSG [72]. The reversibility of the target PTMs is the key to this concept. The specifically reduced and tagged proteins or peptides can be subsequently enriched, thus improving the overall coverage of modified proteins significantly. Common concerns of the selective reduction and tagging strategy are potential false identifications caused by potential incomplete alkylation of free thiols and non-specific reduction of targeted PTMs. Evaluation of these critical steps should be considered during method development and optimization.

**Figure 4:**
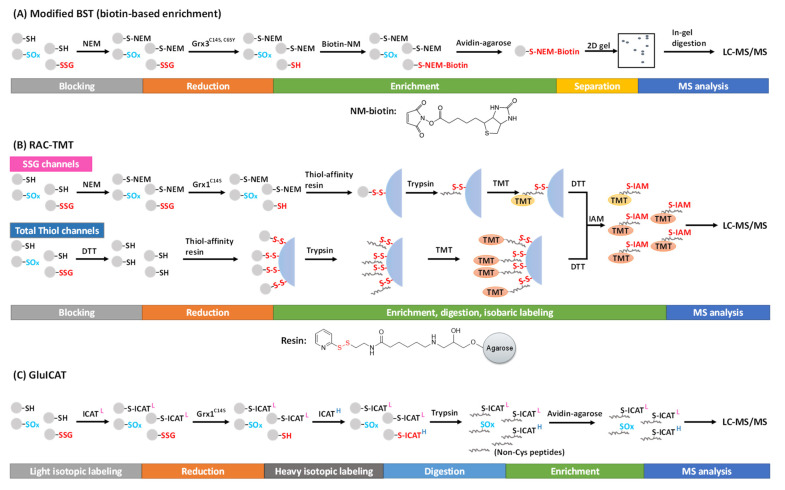
Proteomic approaches for SSG detection and quantification using the selective reduction and tagging strategy. (**A**) Modified BST, (**B**) RAC-TMT. and (**C**) GluICAT approches.

To selectively target SSG, highly specific glutaredoxin (Grx), such as Grx1^C14S^ and Grx3^C14S, C65Y^ from *E. coli*, has been used for the deglutathionylation reaction [72,73,74]. Lind et al. first employed the modified BST to enrich and detect SSG specifically reduced by the mutant Grx3 in the presence of GSH, NADPH, and glutathione reductase (GR) [72]. Free thiols are first alkylated by NEM, followed by the reduction of SSG by Grx3. The resulting nascent free thiols are then alkylated using, NEM-biotin, which can be captured by avidin affinity chromatography (Figure 4A). The captured proteins can be eluted, separated, and visualized on a gel, and further processed for MS-based proteomics analysis. Using this method, 43 novel SSG protein targets were identified including chaperones, cytoskeletal proteins, cell-cycle regulators, and metabolic enzymes in cultured human endothelial-like cells [72]. In addition, this method also allowed visualization of SSG in fixed cells by using *N*-(3-maleimidylpropionyl) biocytin and streptavidin-FITC [75]. However, these biotin-based methods were generally ineffective in site-specific SSG identifications.

Alternatively, the Qian group introduced a resin-assisted capture (RAC) technique for enriching thiol-containing proteins or peptides [50,76], which was later adapted for SSG proteome profiling [8,73,77]. In this workflow, following the blocking of free thiols by NEM, protein SSG is selectively reduced by Grx1^C14S^ in the presence of GSSG, NADPH, and GR. The nascent protein thiols are then captured by a thiol-affinity resin, which contains a 2-thiopyridyl disulfide functional group to react with thiol-containing proteins via thiol-disulfide exchange, forming a mixed disulfide bond. The captured proteins can be eluted by DTT for Western blot or proteomics analyses. The stable covalent capture allows for on-resin protein digestion and subsequent multiplexed isobaric labeling (e.g., with tandem mass tags, TMT). Then, the TMT-labeled, Cys-containing peptides can be eluted from the resin by DTT (Figure 4B). This RAC-TMT approach offers several notable advantages. First, the covalent binding of Cys-containing proteins or peptides on the resin permits harsh washing conditions, thus allowing a high enrichment specificity (i.e., typically >95% of final peptides containing cysteines). Second, the multiplex quantification design enables not only comparative quantification of SSG across different biological samples, but also stoichiometric estimation of SSG occupancy at the Cys site level through incorporating both SSG channels and total thiol channels in the same TMT plex experiment. The total thiol samples are prepared with the reduction of all reversibly oxidized thiols by DTT (without initial blocking of the free thiols), thus representing the sum of all thiols on each given Cys site (except irreversible modifications such as SO_2_H and SO_3_H) (Figure 4B). Thus, the SSG occupancy at each Cys site can be calculated based on the ratio of TMT reporter ion intensities from the SSG channels to those of the total thiol channels [77,78]. The quantification of individual PTM occupancy at the site-specific level is a critical aspect to understand potential functional consequences of PTMs, which was often overlooked in many current studies. Interested readers are redirected to previous reviews on stoichiometric quantification in redox proteomics [79,80].

RAC-TMT and similar approaches have been applied to investigate protein SSG changes under physiological and pathological conditions in different cell types and tissues [8,77,78,81,82]. For example, in a recent study of redox regulation in hyperoxia-induced lung injury, over 7600 SSG sites were quantified by RAC-TMT, providing a landscape view of the SSG proteome in the mouse lung [83]. In another study, Duan et al. quantified both SSG and total oxidation occupancies for ~4000 Cys sites under basal conditions in mouse RAW macrophages, revealing a mean occupancy of 4.0% for SSG and 11.9% for total oxidation [78]. Interestingly, it was observed that the average occupancies of SSG and total oxidation for individual subcellular compartments was well correlated with their redox potentials.

Recently, an approach called GluICAT, a modified version of the original oxICAT method [84], also demonstrated stoichiometric quantification of SSG at the site level [85,86]. Using isotopically light or heavy thiol-reactive ICAT, free thiols and Grx1^C14S^-reduced protein SSG are differentially labeled, enriched, and quantified to determine SSG level vs. free thiol level (heavy-to-light ratio) of individual Cys sites (Figure 4C). This method was able to identify 2133 SSG-peptides (~2300 SSG sites) in Leber’s hereditary optic neuropathy (LHON) fibroblasts, where 439 peptides were quantified to show an increased SSG level [85]. One notable limitation of this method is that it quantifies the ratio of SSG vs. free thiols without considering other types of thiol PTMs (e.g., disulfides). Thus, the data need to be carefully interpreted with consideration of this important caveat.

Finally, there are several other approaches including the Cys-reactive phosphate tag (CPT)- TMT [87] or OxiTMT methods [88] that are potentially applicable, but have not yet been demonstrated for SSG analyses.

### 3.3. Chemoselective Probes

The concept of using chemoselective probes to label free thiols either in vivo or in vitro to form protein-SSG analogs is an interesting chemical biology strategy for enabling the characterization of protein-SSG products. In this strategy, glutathione analogs or tagged amino acids (to produce tagged GSH endogenously) were introduced either for in vivo reactions by using membrane permeable probes during cell culture or for in vitro reactions. Glutathione ethyl ester (BioGEE) [89] and GSSG-biotin [90,91] are the first class of glutathione analogs for in vivo and in vitro labeling, and the biotin tag facilitates the enrichment of protein-SSG products. The use of BioGEE or GSSG-biotin is usually accompanied by addition of pro-oxidants to induce oxidative stress, and the resulting protein-SSG products can be identified by Western blot using anti-biotin antibodies or streptavidin-horseradish peroxidase (HRP). The biotin tags also allow capture of glutathionylated proteins by streptavidin agarose beads, which can be released from beads by a reducing reagent (e.g., DTT), thereby cleaving the mixed disulfide bond within the SSG moiety. The eluted proteins can be further analyzed by gel or mass spectrometry [90,92]. Furthermore, Brennan et al. demonstrated the capacity to image biotinylated protein SSG stained with ExtrAvidin-FITC in cells using confocal fluorescence microscopy [91].

**Figure 5:**
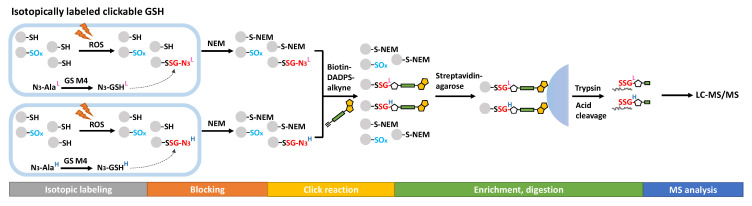
Proteomics approaches for SSG detection using clickable analogs of GSH.

Metabolic labeling with clickable GSH probes has been developed as an alternative approach instead of biotin tags. Clickable GSH [93,94], including azido-GSH, allyl-GSH, and allyl-*O*-GSH, can be biosynthesized in vivo by expressing an engineered glutathione synthetase (GS M4, F152A/S151G), which catalyzes azido-Ala, allyl-Gly, and allyl-Ser respectively instead of the endogenous substrate Gly (Figure 5). A subsequent click reaction (azide-alkyne or tetrazine-alkene bioorthogonal chemistry) can then tag the glutathionylated proteins with biotin or a fluorophore after protein extraction, thus enabling biotin-streptavidin pull-down or fluorescence labeling for detection. This method has been further extended to LC-MS/MS profiling of glutathionylated proteins by using a cleavable biotin-DADPS-alkyne. Biotinylated proteins are enriched by streptavidin agarose and digested by trypsin, then released by an acidic cleavage of the DADPS linker for detection by LC-MS/MS (Figure 5) [95]. By developing light or heavy isotopic labeled clickable GSH, this method was applied to mouse cardiomyocyte cells which quantified 1398 SSG-peptides induced by H_2_O_2_ [96].

It should be noted that the addition of GSSG-biotin to cells in culture itself causes oxidative stress due to an increase in cellular GSSG [91]. On the other hand, intracellularly synthesized clickable GSH may not disturb the cellular GSH/GSSG ratio. Azido-GSH, azido-GSSG, and an azido-GSH-modified model peptide were reported to be efficient substrates of Grx, GR, and GST omega, which does not interfere with the reversibility of the glutathionylation process and GSH/GSSG cycling [93]. However, the application of such probes is limited to living cells that can exogenously express GS M4 and not other types of biological samples, such as tissues. Although the use of chemoselective probes overcomes the potential non-specificity issue of selective reduction and tagging approaches, there are caveats to consider. Introducing these probes themselves is likely to perturb the physiological state and native protein-SSGs are not analyzed.

### 3.4. Global Profiling of the SSG Proteome

Enabled by the advances described above, extensive studies for profiling the SSG proteome in various cells or tissues have been reported. Table 1 lists selected examples of recent studies aiming at the global profiling of the SSG proteome, illustrating the different levels of proteome coverage achieved in various samples using different approaches.

## 4. Functional Roles of S-Glutathionylation

The advances in analytical approaches for SSG characterization has resulted in a continuing expansion of the list of protein SSG modifications. Protein SSG has been reported to cause a decrease (in most cases) or increase in the activity of proteins that are involved in energy metabolism and glycolysis, gene expression, signaling, calcium-dependent channels, apoptosis and autophagy, cell structure and dynamics, and protein folding [48,99,100]. Dynamic changes of protein SSGs were also shown to be prevalent in oxidative stress-associated disease progression, thus providing insights in clinical applications, including discovery of biomarkers, molecular targets for drug development, or therapeutic interventions [48,77,101,102,103,104].

With the identification of SSG modifications, the next important step is to elucidate the structural and functional relevance by orthogonal methods for a mechanistic understanding of their regulatory roles. To pinpoint the regulatory roles of protein-SSG, detailed functional studies will be required. Table 2 lists a set of novel SSG regulated proteins discovered by proteomics followed by functional studies.

GAPDH is one of the most extensively studied redox-sensitive proteins due to its diverse cellular functions in glycolysis, cellular apoptosis, cellular survival, DNA repair, and nuclear tRNA export. The enzymatic activity of GAPDH is inhibited when the catalytic cysteine (in a highly conserved CXXC motif; Figure 1) is glutathionylated [19,113]. Recently, it was reported that glutathionylation of the catalytic cysteine in recombinant plant GAPDH results in partially misfolded proteins and promotes protein self-aggregation to generate insoluble oligomeric particles. The aggregation process can be accelerated by further oxidation of SSG to an intramolecular disulfide bond with the other cysteine in the CXXC motif [110]. It was also found that SSG on C247 is necessary for GAPDH nuclear translocation, while an intra-subunit disulfide bond was observed within the CXXC motif in HEK 293T cells. The nuclear GAPDH binds to Sirtuin-1 (SirT1) and leads to glutathionylation of SirT1 and inhibition of SirT1 deacetylase activity, which subsequently impairs SirT1-p53 interaction and induces apoptosis [114]. Thus, these studies significantly improved the knowledge on the structural and functional consequences of SSG modification and potential pathogenesis related to PTMs on GAPDH [115].

Site specificity and context dependence are important features of SSG regulation. Human Hsp70 SSG was well characterized using multiple techniques including modified BST, NMR, size-exclusion chromatography, and intrinsic fluorescence spectroscopy [105]. Among the five Cys residues, SSGs on C574 and C603 located in the C-terminal α-helical lid of the substrate-binding domain led to unfolding of the lid structure, thus blocking the substrate-binding site and decreasing peptide binding activity. Another example of site-specific regulation is fatty-acid binding protein (FABP5), a protein discovered from RAC-TMT SSG proteome profiling in microphages [9]. FABP5 SSG facilitated fatty acid binding and nuclear translocation under oxidative stress. Although four cysteines of FABP5 were identified with SSG, mutagenesis experiments elucidated that only C127 was required for these events. Molecular docking analysis revealed that C127 was within the ligand-binding pocket, which could perfectly accommodate GSH molecules in both monomeric or dimeric FABP5 [9]. The same study also showed that FABP5 SSG inhibited inflammation in macrophages by binding and activating nuclear receptor PPARβ/δ and its target genes.

Notably, glutathionylation of cryptic cysteines on the elastic protein titin in cardiac muscle was also reported. Cryptic cysteines are buried inside of a protein and not accessible to oxidants under basal conditions. Thus, they remain inert in the folding of the immunoglobulin (Ig) domains in I-band of titin. However, glutathionylation of these residues occurred upon unfolding of Ig domain by heating or mechanical force in the presence of GSSG, which was reversible by Grx [116]. Recently, in vivo characterization of SSG (or oxidation) in unfolded Ig domains suggested a role in regulating human cardiomyocyte passive force [106]. SSG of one Cys (C13585) in a distal Ig domain led to the weakening of titin-based stiffness by stabilizing the unfolded and misfolded domains and promoting aggregation. The novel finding of cryptic Cys modification contrasts common redox-sensitive cysteines with low pK_a_ and good solvent accessibility, further underlining the structure-function relationship in understanding the regulatory role of PTMs.

Interestingly, various PTMs can occur simultaneously on the same protein, group of proteins, or even the same residues, which increases proteome complexity [117]. Crosstalk between PTMs is proposed to contribute to higher order regulation, such as the relationship between protein SSG and phosphorylation. SSG modification in the unfolded Ig domain of titin prevents the refolding of the domain, thus potentially exposing phosphorylation sites. Following thermal unfolding of the Ig domain, glutathionylation of the domain led to higher phosphorylation levels at a single serine residue by Ca^2+^/calmodulin-dependent protein kinase-II delta (CaMKIIδ), compared to the thermal unfolded domain without SSG [106]. The interplay between SSG and phosphorylation was also demonstrated in another work focusing on two myofilament proteins (myosin binding protein C (cMyBP-C) and cardiac troponin I (cTnI)) and titin in human failing hearts [118]. Along with increased levels of hydrogen peroxide and lipid peroxide and decreased levels of GSH, glutathionylation of cMyBP-C, cTnI, and titin were observed in end-stage human failing heart samples. Meanwhile, oxidized protein kinase G (PKG) was found forming dimers and polymers, which were associated with decreased activity, causing titin hypo-phosphorylation. Lower levels of protein kinase A (PKA)-dependent phosphorylation of cMyBP-C and cTnI was also observed in failing heart samples compared to non-failing donors. The dysregulated phosphorylation by PKA is potentially attributed to altered PKA affinity or accessibility of phosphorylation sites following oxidation of vicinal cysteines. This study demonstrates that the contractile dysfunction in human cardiomyocytes is co-regulated by both oxidative stress and phosphorylation, providing therapeutic targets to cardiovascular diseases.

## 5. Conclusions and Perspectives

Protein S-glutathionylation plays a critical role in ROS-mediated signaling and various cellular functions under physiological conditions, while abnormal regulation of SSG can lead to disease development. Recent biochemical and analytical advances have enabled in-depth SSG proteome profiling, and several studies have reported that the SSG modifications occur broadly across all subcellular compartments [77,78,83]. The coverage of the SSG proteome might be further enhanced by adopting recent workflows involving fractionation followed by LC-MS/MS, where a deep coverage of the thiol redox proteome was demonstrated for thiol oxidation [87,119]. In typical quantitative analyses of the SSG proteome between different biological conditions, it is possible to identify 100s or even 1000s SSG sites with statistically significant changes. Besides statistical significance and fold-changes, the stoichiometry information of Cys site SSG occupancy will provide a significant value in determining potential regulatory Cys sites [80]. Future studies investigating the roles of specific redox-regulating enzymes such as GSTP might help to elucidate the broad regulatory landscape of the SSG proteome [81].

One of the notable challenges in the SSG proteome profiling is the difficulty to directly identify in situ SSG moieties by MS approaches, partially due to the bulky tripeptide structure. The SSG moiety on Cys residue can undergo fragmentation in MS/MS mode, leading to neutral losses via cleavage of the peptide bond, carbon-sulfur bond, and even disulfide bond, depending on the dissociation techniques employed by tandem mass spectrometry. A recent LC-MS/MS analysis of in vitro cofilin glutathionylation reported neutral loss of the glutamic acid residue (−129 Da) from the SSG moiety on precursor ions following collision-induced dissociation (CID) [120]. In contrast, electron transfer dissociation (ETD) tended to break the disulfide bond as well as release the whole SSG moiety (−305 Da), which was detectable as a glutathione oxonium ion (308.4 m/z) in the same spectrum. Other neutral losses resulting from cleaved carbon-sulfur bond in Cys residues were observed as well. Peptide identifications of such complicated mass spectra represents a challenge in current database searching algorithms for shotgun proteomics.

Another significant challenge for SSG data interpretation is the complex landscape of the thiol PTMs. In a recent study involving direct detection of multiple types of thiol PTMs, many proteins were observed with ≥4 types of thiol PTMs on single Cys site [70]. These include well-established redox-sensitive protein Cys sites such as the Cys150 of GAPDH with 6 types of thiol PTMs: SSG, SSH, SSSH, SO_2_H, SO_3_H, and a disulfide with C154. This observation of the thiol PTM landscape shed light on the complexity of thiol redox regulation, which might not be exclusively dependent on a particular type of PTM such as SSG or SNO. Further development of techniques that quantify the stoichiometry of multiple types of thiol PTMs will greatly improve our understanding of the details of redox regulation.

The presence of multiple types of redox PTMs on the same protein/residue also implicate distinct regulatory roles or potential functional interplay among them, a largely untapped area in redox biology. Multiple types of thiol PTMs are hypothetically affecting protein functions or activities differently because of the diversity in their chemical structures. In addition, the interplay among distinct redox PTMs can serve as a redox-switch regulatory mechanism on proteins and biological processes. However, the crosstalk between different types of thiol PTMs has been long overlooked due to the lack of methods to characterize them. Recently, Gao et al. reported a switch from SSG to SSH in a group of 250 glutathionylated proteins when diamide-treated pancreatic beta cells were subsequently treated with a H_2_S donor, which restored metabolic flux in glycolysis and TCA cycle under oxidative stress [121]. Competitive actions by SNO and SSG were also reported on the same Cys site of troponin I exerting opposing effects on Ca^2+^ sensitivity in rat fast-twitch muscle fiber [112]. Dynamic characterization of individual or multiple types of thiol PTMs across timepoints and conditions at the site level are expected to facilitate an understanding of the synergetic or competing regulation networks in redox signaling. The crosstalk between redox PTMs and other types of PTMs such as phosphorylation is another interesting area to explore. For example, broad dynamic Cys thiol oxidation was observed during the epidermal growth factor signaling in cancer cells [122], suggesting interplay between redox and phosphorylation PTMs.

The ultimate goal of PTM profiling is to discover and validate novel functional PTM regulators including SSG. While MS-based proteomics can provide large-scale and broad characterization of thiol PTMs, functional studies that investigate the impact of these PTMs on protein structures and activities is a labor intensive and slow process. There is a possibility that advanced computational approaches to integrate these PTMs sites such as SSG can help to elucidate functional significance of SSG in more rapid fashion. One possible approach is to map the PTM sites in protein structures so that their structural localization can inform their effects on protein functions. With the recent advancement of protein structure prediction, AlphaFold 2 now can provide highly accurate predictive structures that are close to experimentally resolved ones [123]. The AlphaFold database now has predicted structures of almost all proteins available, with more than 200 million proteins from about one million species [124]. One can easily map the quantified SSG modification changes to proteins structures [125] and calculate the relative positions of the modified site to the protein surface or specific domains. These analyses can help infer the possible perturbations from SSG on proteins structure, function, or activity. Besides the static structural analysis, it is also promising to study the function of SSG sites using molecular dynamics simulations or Monte Carlo sampling of the conformation or energy landscapes [126,127,128]. Structure and molecular dynamics analyses can facilitate prioritization of functionally important SSG sites and guide the design of validating experiments.

Finally, other forms of GSH-mediated PTMs besides SSG were also reported and may involve in regulatory events. For example, it has been observed that β-elimination of serine, threonine, and cysteine in proteins or phosphoserine and phosphothreonine result in dehydro-amino acids, such as dehydroalanine (Dha) and didehydrobutyrine (Dhb) [129]. The Dha/Dhb can react with GSH via a Michael addition reaction to form an irreversible carbon-glutathionylation (C-SG) modification. The C-SG modifications have been reported in aging tissues [130]. More recently, the protein C-SG was reported on kinases such as MEK1 catalyzed by LanC-like enzymes (LanCLs), which inactivated the kinase activity [131]. The addition of glutathione to Dha/Dhb in proteins can be catalyzed by LanCLs, thus forming CSG. Interestingly, the damage product MEK1-Dha was observed to be aberrantly activated, and LanCL-catalyzed CSG was proposed as a repair mechanism for protein damage physiologically [131].

## Figures and Tables

**Table 1:** Selected MS-based profiling studies of the SSG proteome.

Biological System	Method	Coverage of the SSG Proteome	Experiment Condition	Significance	Ref.
Mouse lung	RAC-TMT	~7600 SSG sites.	Hyperoxia vs. basal; Neonatal wild-type vs. overexpression (β-ENaC)	Landscape view of SSG-modified proteome in mouse lung and the impact of hypoxia on the SSG proteome	[83]
RAW 264.7 mouse macrophages	RAC-TMT	Occupancy for 4099 SSG sites	Basal condition	Proteome-wide quantification of both SSG and total oxidation occupancy under physiological conditions, revealing cellular compartmentation of redox homeostasis.	[78]
Mouse skeletal muscle	RAC-TMT	Occupancy for >2200 SSG sites and changes due to muscle fatiguing	Gastrocnemius muscle with and without fatiguing contractions	Increased muscle protein SSGs identified following a single bout of fatiguing contraction	[77]
HL-1 cardiomyocyte	Clickable GSH	1398 SSG-peptides in isotopic duplex experiment	H_2_O_2_ treatment (1 mM)	In vivo isotopic tagging of protein SSGs for direct enrichment and quantitative detection; Validation (by Western blot and site mutation) of two structural proteins of interest.	[96]
Mouse liver	Modified RAC-TMT	724 SSG-modified proteins	Basal condition, GSTP-nulled mice	The SSG proteome mediated enzymatically by GSTP	[81]
*Synechocystis* sp. PCC6803	GSSG-Biotin	349 proteins with SSG (protein level enrichment); 145 SSG sites (peptide level enrichment)	Lysate treated with GSSG-biotin	First SSG proteome profiling in cyanobacteria by GSSG-biotin and LC-MS/MS.	[97]
*Streptococcus mutans* UA159	IodoTMT switch strategy	357 SSG sites	Wild-type vs. mutants	SSG profiling in in bacteria and mutants; Site mutagenesis validation for SSG function on a thioredoxin-like protein.	[98]
Human skin fibroblasts	GluICAT	2307 SSG sites	LHON patients vs. healthy controls	Quantify the ratio of SSG and free thiols with heavy or light ICAT	[85]

**Table 2:** List of novel SSG-regulated proteins discovered by proteomics followed by functional studies.

Protein	Cys Site	Biological System	Approaches	Structural/Functional Change	Potential Physiological Consequence	Ref.
Hsp70, human	C574, C603	HeLa cells	Modified BST	Unfolding of the α-helical lid structure; Blocking substrate-binding site; Increasing ATPase activity		[105]
FABP	C127	Primary macrophages (mouse)	RAC-TMT, Anti-GSH antibodies (Co-IP)	Promote fatty acid binding function and nuclear translocation; Active PPARβ/δ	Inhibition of LPS-induced inflammation	[9]
Titin distal I-band (82Ig83 domain)	C13585 (cryptic cysteines)	Mouse hearts	OxICAT, Anti-GSH antibodies (Western blot)	Unfolded domain oxidation; Enhance titin phosphorylation	Decrease titin-based stiffness	[106]
ASC (apoptosis-associated speck-like protein containing a CARD)	C171	Bone marrow–derived macrophages (mouse)	Anti-GSH antibodies (Western blot, proximity ligation assay)	Domain rotation leading to reduced area of CARD-CARD binding interface; Preventing oligomerization	Repress NLRP3 inflammasome activation	[107]
C/EBPβ	C201 and C296	3T3L1 preadipocyte	Anti-GSH antibodies (Western blot)	Decreased interaction with PIAS1; Stabilizing C/EBPβ	Promotion of adipogenesis	[108]
BiP	C41, C420	Multiple myeloma cells	Anti-GSH antibodies	Decreased α-helix and increased β-sheets; Enhancing foldase activity; Decreasing ATPase activity	Proteasome inhibitor resistance	[109]
GAPDH	C149	Arabidopsis thaliana	LC-MS/MS	Enzyme inactivation; Misfolding and destabilized conformation; Inducing aggregation and disulfide bond formation with C153		[110]
SMYD2	C13	H9c2 myocytes	Clickable GSH (Western blot)	Dissociation; Disrupts SMYD2–Hsp90–N2A(titin) interactions	Sarcomere destabilization	[93]
Vimentin	C328		LC-MS/MS	Stabilizing pep2B region of vimentin tetramers; Impaired assembly into long filament		[111]
Troponin I	C134	Skinned mammalian muscle fiber	BioGEE, anti-GSH antibodies, LC-MS/MS	Competitive action with SNO on the same Cys site	Increased Ca^2+^ sensitivity	[112]
Estrogen receptor α	C221, C245, C417, and C447	Primary macrophages (mouse)	Anti-GSH antibodies, LC-MS/MS	Reduced binding potential: receptor density and affinity for 17β-estradiol		[36]
